# Medical Society of the County of Albany

**Published:** 1876-05

**Authors:** 


ART. II.—*Medical Society of the County of Albany.* Semi-monthly meeting Feb. 23d, 1876.

Dr. Henry March, President, in the chair. Dr. F. C. Curtis reported a case of Leucocythemia. The following is an abstract:

D. W., aged 30, native of this county, and by occupation a curer of fish. Had always been healthy in general; when young had rheumatism; had been in a malarious region fifteen or twenty years ago, but had not been affected by it. Began about four years ago to have night sweats without accompanying symptoms—often very profuse, and little affected by treatment. A year ago began to feel dull pain in the left side in region of spleen. A few months later the side was protruding, and examination showed the spleen greatly enlarged, reaching to the median line. There was little change in the symptoms excepting a degree of progressive weakness until last July, when for a time he experienced great dizziness. In October profuse diarrhœa set in, which lasted till death. This appeared to have the effect to reduce the size of the spleen, as it diminished greatly. He had dyspnœa and symptoms of heart weakening, which at one time were very urgent. There was congestion of the lungs and a mitral systolic murmur. Thrombosis of popliteal vein occurred on left side, and not long after on right. Examination of the blood showed a large excess of white globules. He began finally to grow stronger although the diarrhœa still continued, no effort being made to check it, as tympanitis and distress always ensued, and he finally was able to sit up most of the day. Cod-liver oil and bitter tonics comprised the treatment. Iron was not borne. For several weeks he appeared to be improving in all respects, when pleurisy set in, and he died ten days after. Post mortem examination showed a spleen having a size of 10 inches by 7½ by 5. The liver was also much enlarged and apparently fatty. Kidneys large. The right pleura was filled completely with serum. The blood was clotted, some of the clots being colorless, but most of the blood having a mahogany color. A point of chief interest in the case is the rapid and permanent reduction of the spleen in size after profuse diarrhœa set in, and also the benefit evidently following the use of cod-liver oil in building him up and possibly affecting his disease specifically.

Dr. Hannon said he thought Leucocythemia a very questionable disease. He would ask three questions: where the white corpuscles were made, how they got into the circulation, and how they destroyed the red globules?

Dr. Curtis said that if the origin of the white globules was a point at issue, it is held by many that the spleen is their starting point, and from these they pass into the circulation, He did not think that they destroyed the red globules.

Dr. Munson asked if there was any difference in the size of the leucocytes, as compared to the normal size, and also if the sight was interferred with.

Dr. Curtis said that he did not know if there was any difference in the size. The sight was not impaired.

Dr. Hannon enquired what caused death?

Dr. Curtis said that he did not understand the question. The white corpuscles destroy life by not being able to support it. The red globules are the life-supporting ones.

Dr. Hannon asked if the enlarged spleen might not have originated from miasm?

Dr. Curtis said that did not constitute Leucocythemia.

Dr. Munson said he thought it an interesting fact that Leucocytes did not emigrate into other tissues, but remained in the blood.

Dr. James S. Bailey remarked that he was much interested in the case and in the post mortem appearances. He saw enough to account for death. This was the second case reported before the society since its organization. Dr. Myron Davis had reported one case seven years before. In regard to the size of the spleen he thought Dr. Van Derveer had one in his office which was larger.

Dr. J. M. Bigelow reported a case of tobacco poisoning. He said: August 28th, 1875, I was hastily summoned to attend Mr. T. S., aged 26, clerk, single, who had been suddenly seized with a convulsion while walking with a friend on the street. He was stricken without premonition, and was conveyed to a neighboring store, where I first saw him. His skin was very pallid and presented an anæmic appearance; his features were pinched and contracted; his pulse was irregular; for a few seconds it was very rapid, then quite slow and labored; sometimes thready and hurried, at others retarded and intermittent. At one time the pulse counted 136 to the minute, and in a short time thereafter sank to 38 per minute. His heart beat very irregularly; this was evinced by palpitative, tremulous motion of the organ, especially when lying upon the left side. The sounds of the heart were muffled, and seemed to almost run into each other, indicating that variety of irritable heart recently mentioned by Dr. Adams inthe *Lancet.* The temperature ranged from 98° to 99.5° F. His lips were pale and bloodless; eyes staring; pupils dilated. He complained of great pain and distress in the left side of the chest, especially around the precordial region. Suffered great dyspnœa, drew long sighs, made a gulping effort at emesis, had hiccough, and cold perspiration, and presented great physical and nervous prostration. These symptoms were rapidly succeeded by clonic convulsions, which produced great muscular agitation, particularly of the extremities. The teeth were shut together, hands tightly clinched; the legs flexed and extended in rapid alternation. With the cessation of these spams, the patient complained of anesthesia, more or less complete, especially of the left side of the extremities and of the tip of the tongue; also spoke of excessive languor and nervous tremor.

These convulsions were not attended by any loss of consciousness, for in their cessation he would repeat our conversation and assure us that although he knew what was occurring near and around him, yet he was powerless to help himself or to control the excessive muscular perturbations. It was during the first visit that I noticed, after the transit of the convulsions, a cataleptic condition of the arms and legs. If a leg or arm was extended or flexed or uplifted, it would retain that position for at least five mintes, or until it was reduced to a more comfortable position This condition passed off and was succeeded almost immediately by hysteric tremors, convulsive twitchings of the flexor muscles of the whole body, accompanied by an agonized apprehension of some rapidly approaching physical catastrophe, the result of which would be death. He would clutch the arm of any by-stander and beseech him to save his life, to relieve him from the great precordial distress and threatening suffocation. This fear was in some subsequent attacks the cause of prolonged mental and bodily excitement. Conversation, rapid walking or any violent motion of the attendants would provoke these spasmodic attacks and produce great nervous irritability. His disposition, from that of amiable, became fretful and pevish. At the time of the first attack there was administered hypodermically gr. ¼ sulph. morphia and prescribed.

℞. Bromide Potass. gr. x.
Ammon. Carb. gr. v.
Aquæ ℥ ii.

Repeat every two hours. He was removed to his home when I saw him again on the 29th of August. On careful enquiry I learned that this attack was the third within a year, each attack occurring at the commencement of each six months. Mr. S. had smoked tobacco since he was twelve years of age, some days using as many as ten cigars, and often substituted for luncheon three or four strong ones. He had little or no appetite most of the time; was pale and cadaverous, languid and weak; ate but little and at irregular hours; but from his rise in the morning to his retirement at night, he was never without his "weed." He was restless; would start and jump in his sleep; had become very irritable in his disposition, and greatly enfeebled. A searching scrutiny of his family history failed to elicit any trace of epilepsy or other nervous disorder, and with regard to himself, he denied any other sickness than the present. The above treatment was continued until the 1st of September, when a tonic of the elixir Ferri Strych. et Quin. ʒi after meals and Pot. Bromide gr. xv at bedtime were prescribed. He had resumed his occupation and had so much improved that I ceased visiting him, after leaving some directions about his diet and general health and interdicting tobacco in any form. I left him not altogether clear in my mind as to the cause of the singular symptoms he had presented. On September 5th, 1875, I was again summoned to him. He was under the influence of the "worst fit he had ever had," as the messenger said. On arrival at the house of the friend he was visiting, I found him reclining on the lounge, presenting the appearance in speech and looks of an acute maniac. At one moment, seizing his friends, he would implore them to save him from some indescribable "something," which was stifling him; the next moment he fell into the most violent convulsions I have ever witnessed. He presented the same symptoms in rapid succession as have been previously narrated, excepting that his fear of death had become a mania. On enquiry I found that he had been quite fretful for a day or two previous, neglecting his meals, and smoked from eight to ten cigars daily. I prescribed,

R. Ammonia,
Quinia,
Zinci Valerianatis aa gr. j,
Simple Syrup dr. i, to be given every three hours,

R. Pot. Bromide gr. x,
Tr. Aromat Comp. dr. i,
Simple Syrup dr. ij, to be given every six hours.

On the 6th of September he was much better, but still suffered severely from hysteric and ataxic tremors of the limbs and arms; also complained of great distress in the cardiac region which would occasionally precipitaate a transient convulsive agitation of the whole body. He could hardly sit upright; called my attention to the great numbness of the extremities and of the tongue; he felt cold and chilly. Continued the same treatment. On the 7th he had so far improved as to be able to sit up in an easy chair; slept well and had no return of the spells for some four hours. On the 8th Dr. Howe was called in consultation, and confirmed the diagnosis of Tobacco Poisoning, and the treatment as given. From this time onward preparations of Strych. and Iron, good diet, fresh air and moderate exercise were enjoined. Also Pot. Bromide in gr. x doses twice daily in combination with Tr. Aromat Comp. ʒi doses. On the 11th he went into the country, and returned on the 30th, fully restored in health, and better than he had been for years. Since that time, on three occasions, he has smoked from four to six cigars in succession, followed by a return, with greater or less violence, of the symptoms. He has since given up entirely the use of tobacco, and now enjoys the best of health.

Connected with the above example of the evil effects of the abuse of tobacco, it may be interesting to note a few other results which have been mentioned as attendants of tobacco poisoning.

Owing to the enormous consumption of tobacco in France and the alarming increase of nervous disorders in the empire, Napoleon, in 1861, called the attention of the Academy of Medicine at Paris to the subject. A scientific statician, with an imperial commission, was empowered to collect facts and data for a report. The result showed that whereas the annual tobacco tax from 1812 to 1862 was 28,000,000 francs, and that in the hospital there were 8,000 paralytics and insane, in 1862 the tax had risen to 180,000,000 francs and the number in the hospitals increased to 40,000 paralytics and insane. A commission was then appointed to enquire into the influence of tobacco on the human system. The report stated that a large number of the diseases of the nervous system and of the heart noticed in the cases of those affected with paralysis or insanity were regarded as the sequence of excessive indulgence in the use of tobacco. M. Jolly said that "Tobacco seems primarily to act upon the organic nervous system, depressing its functions, such as the nutrition of the body, circulation of the blood, and the number of the red corpuscles in the blood." He further states that "tobacco produces weakness of the brain and spinal marrow." Attention was also called to the bad digestion, benumed intelligence and clouded memory of those who used tobacco to excess." Dr. Occaisue reports that "in twenty-two out of thirty-three case of tobacco smokers there were injurious disorders of the circulation, 'bruit de souffle' in the neck, palpitation, disorders of digestion, cloudiness of intellect, &c. In a majority of cases there was diminution of the red corpuscles of the blood and epistaxis, disturbed sleep and vertigo." Dr. B. W. Richardson observes "that smoking produces disturbance of the blood of the stomach, heart and brain, of the organs of sense, and of the nervous filaments of the sympathetic and organic nerves." Again he states that "tobacco smoking arrest oxygenation of the blood, and thus interferes with the full development of the structures of the body, especially in the young." Dr. Hutchinson, of London, alsomentions Amaurosis. Dr. Derby, Assistant to Prof. Von Graffe, notes color blindness as an occasional sequence to tobacco smoking.

Whether the convulsions we have noticed are due to Anæmia of the brain and of the blood, or the result of more or less permanent derangement of the organic system of nerves, is a question of vital interest to all admirers of the fragrant Havana.

Dr. Stonehouse said he had seen one case of acute mania which was produced by excessive tobacco smoking. The patient recovered on quitting its use.

Dr. Hannon said he did not think tobacco had a stupefying effect on the mental faculties, for some of the finest literary efforts have been brought forth after considerable smoking.

Dr. James S. Bailey said that he had experienced something of the evil effects of the weed, but to such an extent as spoken of in Dr. Bigelow's paper. He was happy to say that he used it no longer.

Adjourned.

